# Association of objective sedentary behaviour and self-rated health in English older adults

**DOI:** 10.1186/s13104-019-4050-5

**Published:** 2019-01-11

**Authors:** Jason J. Wilson, Nicole E. Blackburn, Rachel O’Reilly, Frank Kee, Paolo Caserotti, Mark A. Tully

**Affiliations:** 1 Centre for Public Health, School of Medicine, Dentistry and Biomedical Sciences, Queen’s University Belfast, Belfast, UK; 2 UKCRC Centre of Excellence for Public Health, Belfast, UK; 3 Active Belfast, Belfast Health Development Unit, Public Health Agency, Belfast, UK; 4 Department of Sports Science and Clinical Biomechanics, Syddansk Universitet, Campusvej 55, 5230 Odense M, Denmark; 5 Institute of Mental Health Sciences, School of Health Sciences, Ulster University, Newtownabbey, UK

**Keywords:** Sedentary behaviour, Accelerometer, Self-rated health, Older adults

## Abstract

**Objective:**

Reducing sedentary behaviour (SB) might improve the health of older adults. However, we know little about how objectively measured SB impacts on self-rated health in older adults. We aimed to explore the associations between objectively measured SB and self-rated health in English older adults.

**Results:**

A random sub-sample of older adults (≥ 65 years old) from the 2008 Health Survey for England wore an ActiGraph GT1M accelerometer for 7 days. Self-rated health was measured using an item from the General Health Questionnaire. Linear regression and analysis of covariance were used to test the associations between percentage time spent in SB and mean daily minutes in SB and self-rated health (very good/good; fair; bad/very bad), adjusting for covariates. Valid accelerometry datasets were returned by 578 individuals. Significant negative associations between percentage time and mean daily minutes in SB and self-rated health were found. In particular, individuals spending reduced percentages of time being sedentary had higher self-rated health. In conclusion, SB appears to be associated with self-rated health in older people independently from MVPA. If longitudinal research could determine how changes in SB influence self-rated health as individuals’ age, this might be an important lifestyle variable to target for health improvement.

## Introduction

Physical activity (PA) promotes healthy ageing while inactivity is associated with elevated risks of chronic disease and all-cause mortality [1, 2]. Some research suggests that regardless of levels of objectively assessed moderate-vigorous physical activity (MVPA), time spent in sedentary behaviour (SB) is an independent risk factor for ill health, morbidity and mortality [3–6]. Although a recent large meta-analysis suggests these risks are attenuated with 60–75 min of daily MVPA [7]. Inactivity refers to not meeting MVPA guidelines [2], while SB has been defined as “any waking behaviour characterised by an energy expenditure of ≤ 1.5 metabolic equivalents while in a sitting or reclining posture” [8]. Therefore, irrespective of whether an individual meets the MVPA guidelines, prolonged periods of SB are likely to compromise health [3, 9, 10].

The UK population is rapidly ageing, with 2015 figures suggesting there were over 11.6 million (17.8%) aged ≥ 65 years old [11]. Older adults are the most sedentary population group [12], hence they are at higher risk of developing various chronic health conditions. Most intervention studies conducted in older adults have focused on increasing MVPA [13, 14]. Interestingly, older adults who are either physically active or non-sedentary both exhibit improved functional fitness [15]. Thus, targeting sedentary lifestyles may provide an alternative approach to improving health outcomes [16].

It is important to monitor and assess population health status trends [17]. Self-rated health measures are extensively used in public health research [17–19] and are generally accepted as valid measures of health status in population studies, with lower ratings associated with increased morbidity and mortality [20–22]. Self-reported health assessments reflect an individual’s perception of their own health status and are usually consistent with objective health measures [18, 22]. SB can be measured using subjective (questionnaires) and objective tools (accelerometers) [23]. High levels of SB appear to be related to lower self-rated health [24–27]. However, most studies have used subjective SB tools which are subject to misclassification bias and recall bias; particularly in older adults [28]. One study has explored the association between objectively measured SB and health status in older adults [25]. However, this study sampled older adults living in retirement communities who may not represent the general older adult population and assessed health through numerous questionnaires. Single questions to assess health status are more practical, easier to interpret and likely to be used more widely in both research and clinical practice [29].

This study aims to explore the associations between objectively measured SB and self-rated health in English older adults using data from the Health Survey for England (2008). The hypothesis is that higher levels of SB will be associated with reduced self-rated health.

## Main text

### Data collection

The Health Survey for England is a repeat cross-sectional survey designed to monitor trends in health and health-related behaviours in a nationally representative and random sample of adults and children living in private English households. The Oxford Research Ethics Committee (reference 07/H0604/102) approved the survey. The 2008 report particularly focused on PA and fitness. A nurse visited each consenting participant to fill in questionnaires. In addition, a random sub-sample wore an ActiGraph GT1M accelerometer on the waist using an elastic belt for seven consecutive days during waking hours; allowing for a cross-sectional study. More details on recruitment methods and sampling strategy are contained in the main report [30]. Standardised outcome variables were produced from raw accelerometry data using KineSoft (3.0.98). Each participant’s dataset was deemed valid if the accelerometer was worn ≥ 600 min/day for at least 4 days [31] with no minimum weekday/weekend day criteria. Exclusion criteria included being immobile/wheelchair bound, having a latex allergy, recent abdominal surgery, failure to provide at least four valid days or were otherwise deemed unsuitable to wear the ActiGraph. Percentage time spent in SB and mean daily time in SB (i.e. < 200 counts per minute from the vertical axis) were used as primary outcome measures with sleep-time being excluded.

Self-rated general health was measured using the relevant item in the 12-item General Health Questionnaire (GHQ-12) [32]. Participants were asked to rate their health using the following: very good, good, fair, bad and very bad. Small participant numbers in certain groups meant these were collapsed to three groups (i.e. very good/good, fair and bad/very bad).

### Statistical analysis

Summary data are reported as mean (standard deviation) unless otherwise stated. Characteristics of respondents who returned a valid accelerometer reading were compared with those who did not using basic descriptive statistics (independent samples t-tests and Chi square). Linear regression analysis (adjusted for covariates) was used to assess the association of percentage time spent in SB (Model 1) and daily time spent in SB (Model 2) and self-rated health. Analysis of Covariance was used to compare the percentage and mean daily time in SB between three groups of participants according to their self-rated health, with Bonferroni correction. Age, sex, body mass index (BMI; kg/m^2^), education (university degree, secondary qualification or no qualification), total household income (£), urban/rural classification (urban, suburban/town or village/hamlet/isolated dwelling), smoking status (never smoked, occasional smoker, regular smoker or current smoker), current alcohol drinker (yes/no), presence of long standing health conditions (yes/no) and mean daily minutes in MVPA (i.e. > 2019 counts per minute) were used as covariates. Analysis was conducted using SPSS v22 (IBM Corporation, USA), with statistical significance set at p < 0.05.

### Results

From the original dataset containing 1250 older adults, 578 returned valid accelerometry datasets. Participants who returned a valid accelerometry dataset were significantly younger (p < 0.001), more educated (p = 0.016), were less likely to be female (p = 0.006), more likely to currently drink alcohol (p = 0.005) and had higher self-rated health (p < 0.001) compared to those who did not return a valid dataset. No differences in terms of BMI (p = 0.867), total household income (p = 0.082), urban/rural classification (p = 0.316), smoking status (p = 0.052) and presence of long standing health conditions (p = 0.363) were identified between these groups. Table 1 provides the characteristics of the valid sample.

**Table 1:** Descriptive statistics for included participants with valid accelerometer data (n = 578)

Variables (n = 578)	Mean (SD)
Daily time in sedentary behaviour (min/day)	617.61 (83.41)
Percentage of time in sedentary behaviour (%)	75.47 (9.45)
Daily time in moderate-vigorous physical activity (min/day)	14.97 (19.73)
Percentage of time in moderate-vigorous physical activity (%)	1.79 (2.32)
*Self-rated health, n (%)*	
Very good/good	367 (63.5)
Fair	158 (27.3)
Bad/very bad	53 (9.2)
Age (years)	73.69 (6.38)
*Sex, n (%)*	
Males	274 (47.4)
Females	304 (52.6)
Body mass index (kg/m^2^)*	28.13 (4.84)
*Urban/rural classification, n (%)*	
Urban	449 (77.7)
Suburban/town	46 (8.0)
Village/hamlet/isolated dwelling	83 (14.4)
*Smoking status, n (%)*	
Never smoked	239 (41.3)
Occasional smoker	35 (6.1)
Regular smoker	249 (43.1)
Current smoker	55 (9.5)
*Current alcohol drinker, n (%)*	
Yes	459 (79.4)
No	119 (20.6)
*Long standing illness, n (%)*	
No condition present	401 (69.4)
Condition present	177 (30.6)

* n = 534

** n = 460

Before adjustment for covariates, there was a negative trend between self-rated health and percentage time and daily time in SB in the sample of 578 participants (Table 2). Due to missing data, the sample size for the analysis reduced to 435 participants. In Model 1 using percentage time in SB, a significant negative association was found (p = 0.003; adjusted r^2^ = 0.410) (Table 2). In Model 2 using daily time in SB, a significant negative association was also found but to a lesser extent (p < 0.001; adjusted r^2^ = 0.177) (Table 2).

**Table 2:** Sedentary behaviour in categories of self-rated health before and after adjustment for covariates

Self-rated general health (n = 578)	Mean percentage time spent in sedentary behaviour (%)	Mean daily time in sedentary behaviour (min)
*Before adjustment for covariates [mean (SD)]*		
Very good/good (mins/day), n = 367	73.19 (8.79)	602.41 (77.89)
Fair (mins/day), n = 158	78.45 (9.40)	639.23 (87.86)
Bad/very bad (mins/day), n = 53	82.40 (8.21)	658.36 (80.30)
*After adjustment for covariates [mean (SE)]*		
Very good/good (%), n = 277	73.89 (0.46)	605.76 (4.80)
Fair (%), n = 119	77.44 (0.71)	634.98 (7.39)
Bad/very bad (%), n = 39	79.57 (1.23)	637.28 (12.75)

After adjustment, individuals who rated their health as very good/good spent 3.56% [95% confidence interval (95% CI) − 5.72 to − 1.41] and 5.66% (95% CI − 8.92 to − 2.40) proportionally less time in SB compared to fair and bad/very bad (both p < 0.001) (Table 3). Considering daily times, individuals rating their health as very good/good spent significantly less time in SB than fair (− 29.23 min/day; 95% CI − 51.62 to − 6.83) but not bad/very bad (31.52 min per day; 95% CI − 65.38 to 2.34).

**Table 3:** Pairwise comparison of sedentary behaviour between groups after adjustment for covariates

Self-rated general health (n = 435)		Mean difference	95% CI for difference	p-value
*Percentage time spent in sedentary behaviour (%)*				
Very good/good (n = 277)	Fair (n = 119)	− 3.56	− 5.72 to − 1.41	< 0.001*
	Bad/very bad (n = 39)	− 5.66	− 8.92 to − 2.40	< 0.001*
Fair (n = 119)	Bad/very bad (n = 39)	− 2.10	− 5.37 to 1.17	0.371
*Daily time spent in sedentary behaviour (min/day)*				
Very good/good (n = 277)	Fair (n = 119)	− 29.23	− 51.62 to − 6.83	0.005*
	Bad/very bad (n = 39)	− 31.52	− 65.38 to 2.34	0.077
Fair (n = 119)	Bad/very bad (n = 39)	− 2.30	− 36.26 to 31.67	1.000

* p < 0.05 using Bonferroni method

### Discussion

This is the first study to explore associations between objectively measured SB and self-rated health in free-living older adults. A significant negative association between SB and self-rated health was found after accounting for important covariates.

These results support other research exploring associations between SB and self-rated health in older adult populations. For example, a cross-sectional study of older adults in Latin America found that those in the highest sitting time categories were more likely to report poor self-rated health [24]. This finding was replicated in other American older adult populations [25, 26]. Davies and colleagues [27] have demonstrated that after controlling for confounders, increased screen-time in older adults exacerbates the impact of low MVPA levels on the likelihood of self-rating their health as poor or fair. A large prospective cohort study in adults aged ≥ 45 years old measured self-rated health as a confounding variable when exploring the association between sitting time and all-cause mortality [33]. The authors highlighted that individuals with lower self-rated health appeared to be the most sedentary (without confounder adjustment). More research has been conducted on PA, with studies showing that better self-rated health among adults appears to be related to a sufficiently active lifestyle [34–37].

These associations are unsurprising considering that SB has consistently been shown to be an independent risk factor for physical and psychological health conditions such as type II diabetes, cancer and anxiety [3, 9, 38, 39]. Qualitative research has also explored the individual motivators and impacts of SB. Individuals have highlighted how prolonged sitting causes increased pain, stiffer joints and more depressed feelings [40]. Older adults taking part in a SB reduction intervention identified positive physical impacts such as improved chronic pain management, balance and sleep quality with positive psychological impacts including feelings of better health, less perceived fatigue and enhanced concentration [6].

Bailis et al. suggest that self-rated health does not simply involve spontaneous assessments of one’s health status and related practices, but is rather an enduring self-concept (i.e. regulated by efforts to achieve health-related goals) [41]. Additionally, a unified conceptual model has been proposed which describes the reflective process an individual goes through when self-rating their health [21]. One aspect of this reflection suggests that bodily sensations provide important signs of physiological imbalance unique to the individual. Our findings and those from other studies would suggest that SB fits well into this model. For example, a retired individual reflecting on their health status may consider how they were less sedentary while working, compared to now; resulting in increased feelings of stiffness and fatigue. Thus, self-rated health is a complex phenotype that may be variably impacted by different health problems and their functional consequences so these reflections are likely to have an important influence on how individuals rate their current health status [42].

An interesting finding were the different results found when considering percentage time versus mean daily time in SB. After adjustment, percentage time in SB appeared to be a strong predictor of self-rated health whereas daily time in SB gave mixed results. This is an important consideration for future research as participants wear accelerometers for a range of hours across their daily waking time. For example, participant one may be sedentary for 10 h per day versus participant two for 13 h per day. However, participant one may be sedentary for 90% of their waking day versus participant two who is sedentary for 75%. Therefore, it is important to consider SB relatively to wear time.

In conclusion, this study suggests that SB could be a novel and important modifiable lifestyle variable to target for health improvement. For researchers and clinicians, the current study findings are important. Patients providing more negative ratings of their health are likely to spend more time in SB. Therefore, helping such patients’ target reductions in SB may be an important step to take for health improvement. Future research should plan for long-term follow-up in order to determine how changes in SB influence self-rated health as individuals’ age.

## Limitations

Activity monitors were only implemented in the 2008 Health Survey for England and have not been subsequently used. This means a cross-sectional design, meaning causality cannot be established. Combining some of the self-rated health categories was not ideal due to insufficient numbers in certain categories. Almost 54% of the sample was lost due to exclusions of invalid data. The valid sample were younger, more educated and had higher self-rated health; potentially reducing the representativeness of the findings. Missingness of confounding variables from some participants reduced the sample size. It was not possible to impute values due to the extent of missing data. Reporting heterogeneity may also have occured (i.e. participants may have reported health differently depending upon perceptions of their own health) [43]. All potential confounders were possibly not accounted for (e.g. cardiovascular fitness and depression) which have previously been shown to be important predictors of self-rated health [35, 44]. The association also appeared to be non-linear in nature.

An important strength of this study was that SB was objectively measured, unlike many other studies which have used self-report instruments. These are often subject to recall and social-desirability bias. The sample was drawn from a large, representative cohort, and levels of MVPA have been controlled for in the analysis.

## Abbreviations

95% CI: 95% confidence interval
BMI: body mass index
MVPA: moderate-vigorous physical activity
kg/m^2^: kilograms per metre squared
PA: physical activity
SB: sedentary behaviour
SD: standard deviation
SE: standard error
